# Peripherally Administered Y_2_-Receptor Antagonist BIIE0246 Prevents Diet-Induced Obesity in Mice With Excess Neuropeptide Y, but Enhances Obesity in Control Mice

**DOI:** 10.3389/fphar.2018.00319

**Published:** 2018-04-05

**Authors:** Liisa Ailanen, Laura H. Vähätalo, Henriikka Salomäki-Myftari, Satu Mäkelä, Wendy Orpana, Suvi T. Ruohonen, Eriika Savontaus

**Affiliations:** ^1^Institute of Biomedicine, Research Centre for Integrative Physiology and Pharmacology, Turku Center for Disease Modeling, University of Turku, Turku, Finland; ^2^Drug Research Doctoral Program, University of Turku, Turku, Finland; ^3^Unit of Clinical Pharmacology, Turku University Hospital, Turku, Finland

**Keywords:** neuropeptide Y, Y_2_-receptor, BIIE0246, obesity, the metabolic syndrome

## Abstract

Neuropeptide Y (NPY) plays an important role in the regulation of energy homeostasis in the level of central and sympathetic nervous systems (SNSs). Genetic silencing of peripheral Y_2_-receptors have anti-obesity effects, but it is not known whether pharmacological blocking of peripheral Y_2_-receptors would similarly benefit energy homeostasis. The effects of a peripherally administered Y_2_-receptor antagonist were studied in healthy and energy-rich conditions with or without excess NPY. Genetically obese mice overexpressing NPY in brain noradrenergic nerves and SNS (OE-NPY^DβH^) represented the situation of elevated NPY levels, while wildtype (WT) mice represented the normal NPY levels. Specific Y_2_-receptor antagonist, BIIE0246, was administered (1.3 mg/kg/day, i.p.) for 2 or 4.5 weeks to OE-NPY^DβH^ and WT mice feeding on chow or Western diet. Treatment with Y_2_-receptor antagonist increased body weight gain in both genotypes on chow diet and caused metabolic disturbances (e.g., hyperinsulinemia and hypercholesterolemia), especially in WT mice. During energy surplus (i.e., on Western diet), blocking of Y_2_-receptors induced obesity in WT mice, whereas OE-NPY^DβH^ mice showed reduced fat mass gain, hepatic glycogen and serum cholesterol levels relative to body adiposity. Thus, it can be concluded that with normal NPY levels, peripheral Y_2_-receptor antagonist has no potential for treating obesity, but oppositely may even induce metabolic disorders. However, when energy-rich diet is combined with elevated NPY levels, e.g., stress combined with an unhealthy diet, Y_2_-receptor antagonism has beneficial effects on metabolic status.

## Introduction

Neuropeptide Y (NPY), a 36-amino-acid neurotransmitter, plays a well-known role in the regulation of energy homeostasis aiming at energy storage during negative energy balance (Billington et al., 1991; Zarjevski et al., 1993; Sainsbury et al., 1997). In the central nervous system (CNS), NPY nerves densely innervate the hypothalamus, especially the arcuate nucleus (Arc) and the paraventricular nucleus (PVN), which are responsible for regulation of feeding (Chronwall et al., 1985). Additionally, NPY is co-expressed with noradrenaline in central noradrenergic neurons and peripheral sympathetic nervous system (SNS) (Ekblad et al., 1984), where NPY promotes weight gain by inhibiting lipolysis, and stimulating adipogenesis and angiogenesis in the adipose tissue, and is indispensable for stress-induced obesity (Zukowska-Grojec et al., 1998; Bradley et al., 2005; Kuo et al., 2007; Zhang et al., 2014). NPY acts via six G-protein coupled Y-receptors (Y_1_–Y_5_ and y6), which bind also the other members of the NPY family, i.e., peptide YY (PYY) and pancreatic polypeptide (PP) (Silva et al., 2002). Most of them (Y_1_, Y_2_, Y_5_, and y_6_) have been implicated in feeding and in the control of energy homeostasis (Ekblad et al., 1984; Bowers et al., 2004; Yulyaningsih et al., 2014).

Presynaptic Y_2_-auto-receptors regulate the release of NPY in the hypothalamus and SNS. Gut-derived Y_2_-receptor agonist, PYY_3-36_, reduces food intake by inhibiting NPY release via presynaptic Y_2_-receptors in the Arc (Batterham et al., 2002), which is abolished by Arc-specific administration of Y_2_-receptor antagonist (Abbott et al., 2005). Knock-out of hypothalamic Y_2_-receptors in mice leads to hyperphagia and predisposes to obesity (Shi et al., 2010). Similarly, several human single nucleotide polymorphisms (SNPs) in Y_2_-receptor (rs1047214, rs12649641, rs6857715, and rs17376826) are associated with obesity (Lavebratt et al., 2006; Torekov et al., 2006; Siddiq et al., 2007; Hunt et al., 2011). In contrast, extra-hypothalamic, postsynaptic Y_2_-receptors mediate the obesogenic effects of NPY. Knock-out of germline Y_2_-receptors has been shown to reduce body weight and adiposity both on regular chow and on high-fat diet (Soloveva et al., 1997; Sainsbury et al., 2006), and also to improve glucose and cholesterol metabolism of genetically obese and type 2 diabetic *ob/ob* mice (Naveilhan et al., 2002; Sainsbury et al., 2002). Mice with an adult-onset knock-down of peripheral Y_2_-receptors are resistant to diet-induced obesity (DIO) and have improved glucose clearance (Shi et al., 2011). Treatment with a peripheral Y_2_-receptor antagonist has been shown to improve the metabolic status of diabetic rats by decreasing serum cholesterol and triglyceride levels (Liu et al., 2013). Furthermore, inhibition of adipose tissue Y_2_-receptors has been shown to prevent NPY-induced growth of fat mass *in vivo* and pre-adipocyte (3T3-L1) differentiation *in vitro* (Kuo et al., 2007; Rosmaninho-Salgado et al., 2012).

Based on these findings, the obesogenic role of peripheral Y_2_-receptors seems quite convincing and antagonism of peripheral Y_2_-receptors a plausible anti-obesity drug strategy. To this end, we aimed to test the anti-obesity effects of chronic treatment with a specific Y_2_-receptor antagonist with a potential clinical application to be obesity and metabolic disorders due to NPY excess induced by chronic stress or genetic factors (gain-of-function polymorphisms) (Karvonen et al., 1998; Ding et al., 2005; Masoudi-Kazemabad et al., 2013). In order to study this, transgenic mice overexpressing NPY in noradrenergic neurons (OE-NPY^DβH^) and wildtype (WT) control mice were subjected to chow or DIO with Western type diet and treated with peripheral Y_2_-receptor antagonist, BIIE0246, which is highly selective for its receptors (Doods et al., 1999) and unable to penetrate the blood-brain barrier (Brothers et al., 2010). OE-NPY^DβH^ mice present a genetic obesity model with the metabolic syndrome-like phenotype and with increased peripheral NPY (Ruohonen et al., 2008; Vahatalo et al., 2015; Ailanen et al., 2017). We hypothesized that Y_2_-receptor antagonism would improve the metabolic status especially in OE-NPY^DβH^ mice and in DIO. As expected, it decreased fat mass gain in an energy-rich environment in OE-NPY^DβH^ mice, but surprisingly impaired the metabolic status in WT mice on both diets.

## Materials and Methods

### Animals

Homozygous transgenic male OE-NPY^DβH^ and WT mice on a C57Bl/6N background were used in the experiments. The transgene construction, delivery to noradrenergic neurons with dopamine-β-hydroxylase *(DβH)* gene promoter (Ruohonen et al., 2008), and the metabolic phenotyping of homozygous OE-NPY^DβH^ mice has been previously published (Vahatalo et al., 2015; Ailanen et al., 2017). The mice were housed in an animal room maintained at 21 ± 3°C with a 12-h light/12-h dark cycle (lights on at 6 a.m.). To study the effect of Y_2_-receptor antagonism in healthy conditions, standard rodent chow (9 kcal% fat, 22 kcal% protein, 69 kcal% carbohydrates, SDS, Essex, United Kingdom) was fed *ad libitum* to OE-NPY^DβH^ (NPY) and WT mice. To study the effect in DIO, Western diet (41 kcal% fat, 17 kcal% protein, 43 kcal% carbohydrates, D12079B, Research Diets, New Brunswick, NJ, United States) was fed for 8 weeks prior to the drug administration, and the groups were named DIO-NPY and DIO-WT. Tap water was freely available. Animals (1-3/group) were excluded from the study if they did not gain weight on Western diet (before habituation body weight gain < 3 g and fat mass gain < 2 g). Animal care was in accordance with the guidelines of the International Council of Laboratory Animal Science (ICLAS), and all the experimental procedures were approved by the Finnish national animal care and use committee.

### Experimental Procedures

Drug treatment was studied at the age of 20 weeks. Prior to treatments the mice were habituated for 2 weeks to the handling stress with daily saline injections (i.p.). Half of the chow-fed mice (*n* = 10–13/group) were treated for 4.5 weeks, and half were sacrificed and tissues collected already after 2-week treatment (*n* = 7–14/group). DIO mice (*n* = 7–12/group) were treated for 2 weeks. (The study protocol is presented in Supplementary Figure 1). Mice received 1.3 mg/kg of Y_2_-receptor antagonist (BIIE0246, Tocris Bioscience, Bristol, United Kingdom) or vehicle (DMSO, Tween^®^ 80) (Fisher Scientific, Fair Lawn, NJ, United States and 0.9% NaCl, 1:1:18, respectively) with daily intraperitoneal (i.p.) injections. BIIE0246 with a dose of 2 mg/kg has previously been used in a study with acute administration (Forbes et al., 2012), which supports rationality of the dose (1.3 mg/kg) being used with repeated dosing in the present study. The half-life of BIIE0246 in mouse is less than 3 h, but markedly longer than the half-life of the other Y_2_-receptor antagonists, thus making it the most suitable Y_2_-receptor antagonist for chronic administration (Brothers et al., 2010).

Mice were weighed twice a week and food intake per cage was measured once a week. Food intake per cage (*n* = 3–6 cages/group) was divided by the number of animals in each cage and presented as an average daily energy intake per cage. Body composition was measured from conscious mice with EchoMRI-700 (Echo Medical Systems LLC, Houston, TX, United States) at the initiation of the Western diet, prior to the habituation and the drug treatment periods, after 2-week drug treatment, and before euthanasia. Each animal was scanned twice and the average values for body fat and lean tissue mass were calculated. Mice were divided into treatment groups based on their body weights, and at the initiation of the drug treatments the body compositions were similar between treatment groups within the same genotype (Supplementary Figure 2). As previously shown, NPY mice had increased body weight and fat mass, and decreased lean mass compared to WT on chow, whereas DIO-NPY and DIO-WT mice had similar body weights and adiposity at the beginning of drug treatment (Ruohonen et al., 2012). Energy intake was similar between groups during the habituation period (Supplementary Figure 2).

At termination, mice were fasted for 3 h and blood glucose was measured from awake animals with the Precision Xtra Glucose Monitoring Device (Abbott Diabetes Care, Abbott Park, CA, United States). Mice were then anesthetized with ketamine (75 mg/kg i.p. Ketaminol, Intervet Oy, Espoo, Finland) and medetomidine (1 mg/kg i.p. Cepetor, ScanVet Oy, Vantaa, Finland). Serum was obtained by cardiac puncture, after which the animal was euthanized by cervical dislocation. Subcutaneous, epididymal, retroperitoneal and mesenteric white adipose tissue (WAT) pads, interscapular brown adipose tissue (BAT) and liver were collected and weighed. Medial basal hypothalamus was isolated with a mouse brain block using a 3-mm section caudal to the optic nerve chiasma. The 3-mm brainstem section extended 2-mm caudal from the hypothalamic section excluding the cerebellum and cerebral cortex.

### Blood Parameters

Serum insulin levels were quantitated with an ultrasensitive mouse ELISA kit (Mercodia AB, Uppsala, Sweden), and serum cholesterol with the Cholesterol Fluorometric Assay kit (Cayman Chemical Company, Ann Arbor, MI, United States) according to the manufacturer’s instructions.

### Liver Histology and Adiposity

In order to detect liver histology, samples were fixed with formalin and embedded in paraffin. Liver morphology and glycogen content were analyzed with standard light microscope from paraffin sections (5 μm) on microscopic slides stained with haematoxylin and eosin (H&E) or Periodic acid-Schiff (PAS), respectively. In order to analyze the hepatic lipids, frozen liver samples were homogenized in PBS with 0.1% NP-40, and centrifuged (2 min at 16 000 rpm). Triglycerides and cholesterol were determined from the supernatant with TR0100 Serum triglyceride determination kit (Sigma-Aldrich, St. Louis, MO, United States) and Cholesterol (Total) CHOD-PAP Kit (Biolabo, Maizy, France), respectively.

### Real-Time qPCR

Total RNA was isolated from tissue samples stored in RNA Stabilization Reagent (RNAlater, Qiagen, Hilden, Germany). Retroperitoneal WAT and BAT were snap frozen in liquid nitrogen without RNAlater. RNAs from BAT and WAT samples were extracted with the Trizol Reagent (Invitrogen, Carlsbad, CA, United States), from liver with RNeasy Mini Kit (Qiagen), from the brain sections with RNeasy Lipid Tissue Mini Kit (Qiagen) and from adrenal gland with Arcturus^®^ PicoPure^®^ RNA Isolation Kit (Applied Biosystems, Foster City, CA, United States). RNA was converted to cDNA with High Capacity RNA-to-cDNA Kit (Applied Biosystems) and SYBR Green (KAPA SYBR^®^ FAST ABI Prism^®^, Kapa Biosystems, Woburn, MA, United States) technique with separate primers (available upon request) was used for quantification. Target genes were quantified with 7300 Real-Time PCR System (Applied Biosystems) relative to the housekeeping gene β-actin (*Actb*) (NM_007393.5) or ribosomal protein S29 (*Rps29*) (NM_009093.2), and formula 2^-ΔΔCT^ was used for calculating the gene expression. Expression levels were presented relatively to the expression levels of vehicle-treated WT mice.

The mRNA expression was analyzed from the samples collected after 2 weeks of treatment both in chow and DIO groups in order to detect the changes in energy metabolism and sympathetic tone induced by drug treatment. *Npy* (NM_023456) and Y_2_-receptor (*Y2r*) (NM_008731.3) mRNA expression were quantitated in adrenal gland, *Npy*, tyrosine hydroxylase (*Th*) (NM_009377.1), pro-opiomelanocortin (*Pomc*) (NM_001278582.1) and *Y2r* expression in the hypothalamus, and *Npy* and *Th* expression in the brainstem. In addition, carboxylesterase 3 (*Ces3*) (BC019198.2), fatty acid binding protein 4 (*Fabp4*) (NM_024406.3), hormone sensitive lipase (*Lipe*), (NM_010719.5) lipoprotein lipase (*Lpl*) (NM_008509.2) and matrix metallopeptidase (*Mmp3*) (NM_010809.2) mRNA expressions were analyzed in retroperitoneal WAT, uncoupling protein 1 (*Ucp1*) (NM_009463.3) mRNA expression in BAT, and glycogen metabolism related genes glycogen synthase (*Gys2*) (NM_145572.2) and phosphorylase (*Pygl*) (NM_133198.2) mRNA expressions in the livers of DIO groups.

### Statistical Analyses

Statistical analyses were carried out using GraphPad Prism 6.0 (GraphPad Software, San Diego, CA, United States). Data are presented as means ± SEM and the results were considered statistically significant at *P* < 0.05. The comparisons between the genotypes and the treatments were analyzed with two-way ANOVA, except for food intake, which was analyzed over time with repeated measures ANOVA. Bonferroni *post hoc* test was performed in case the interaction between treatment and genotype was significant to analyze the treatment effect within each genotype. Correlations were analyzed with the Pearson correlation test, and linear regression analysis was used to test the differences between regression slopes and intercepts.

## Results

### Body Composition

**FIGURE 1:**
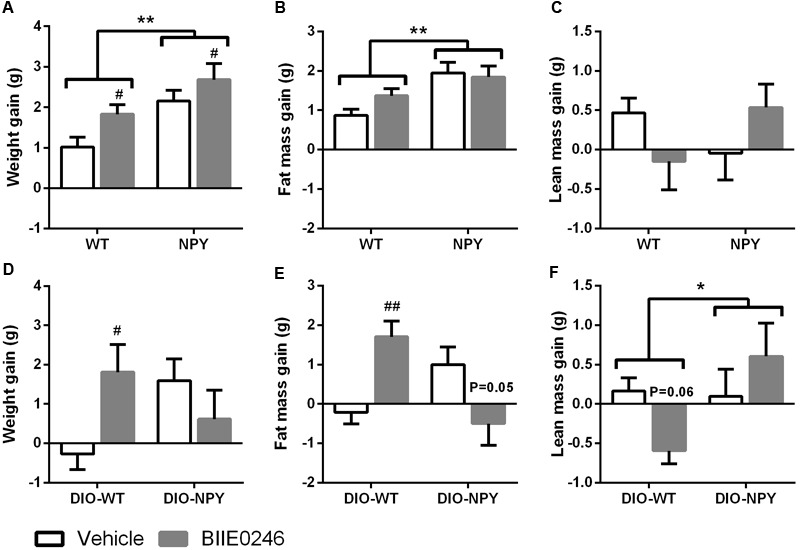
**(A,D)** Body weight, **(B,E)** fat mass and **(C,F)** lean mass gain in OE-NPY^DβH^ and WT mice on chow (*n* = 11–12/group) **(A–C)** or Western diet (*n* = 7–11/group) **(D–F)** treated with Y_2_-receptor antagonist (BIIE0246) or vehicle for 4.5 or 2 weeks, respectively. Values are expressed as means ± SEM. ^∗^*P* < 0.05 and ^∗∗^*P* < 0.01 comparing the different genotypes with two-way ANOVA, and ^#^*P* < 0.05, ^##^*P* < 0.01, *P* = 0.05 and *P* = 0.06 comparing BIIE0246 treatment and vehicle treatment with two-way ANOVA **(A)**, or with Bonferroni *post hoc* test following a significant interaction between treatment and genotype in two-way ANOVA **(D–F)**. White bars, vehicle treated mice; gray bars, BIIE0246 treated mice; WT, wildtype mice on chow diet; NPY, OE-NPY^DβH^ mice on chow diet; DIO-WT, wildtype mice on Western diet; DIO-NPY, OE-NPY^DβH^ mice on Western diet.

First, the effect of BIIE0246 on body composition was studied. On chow diet, genetically obese NPY mice showed increased gain in body weight and adiposity (**Figures 1A–C**). Treatment with BIIE0246 promoted body weight gain in both genotypes after 4.5 weeks (**Figure 1A**), and already at 2 weeks (Supplementary Figure 3). BIIE0246 had no significant effect on fat mass gain (**Figure 1B**). However, there was a different effect of BIIE0246 on lean mass gain between the genotypes (treatment × genotype interaction *P* = 0.05) suggesting that weight gain in NPY (but not in WT) mice is due to lean mass gain (**Figure 1C**).

In DIO, BIIE0246 had different effects on body weight and composition depending on the genotype (treatment × genotype interaction in body weight *P* < 0.05, in fat mass *P* < 0.001 and in lean mass *P* < 0.05). In DIO-WT group, *post hoc* analysis revealed increased body weight and fat mass gain, and a tendency to decreased lean mass gain. In DIO-NPY, BIIE0246 inhibited fat mass gain (*P* = 0.05), and did not change body weight or lean mass gain compared to vehicle (**Figures 1D–F**).

### Food Intake

**FIGURE 2:**
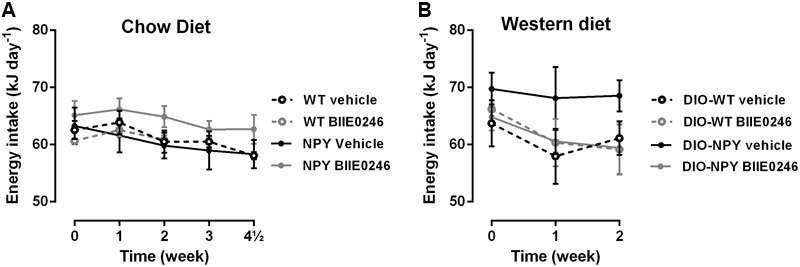
**(A,B)** Energy intake of chow and western diet-fed OE-NPY^DβH^ and WT mice (*n* = 3–6 cages/group) treated with Y_2_-receptor antagonist (BIIE0246) or vehicle for 4.5 or 2 weeks, respectively. Values are expressed as means ± SEM and present an average value per mouse per cage. Statistics is analyzed with repeated measures two-way ANOVA. WT, wildtype mice on chow diet; NPY, OE-NPY^DβH^ mice on chow diet; DIO-WT, wildtype mice on Western diet; DIO-NPY, OE-NPY^DβH^ mice on Western diet.

Treatment with BIIE0246 did not change the energy intake compared to vehicle treatment on chow diet or in DIO (**Figure 2**).

### Blood Glucose and Lipids

In order to elucidate the effects of BIIE0246 on glucose and lipid metabolism, relevant markers in serum and liver were measured in the 4.5-week chow cohort with more pronounced weight effect than the 2-week cohort, and in the DIO cohort. BIIE0246 or NPY genotype had no effect on glucose levels on either diet (**Table 1**). However, serum insulin levels and HOMA-IR index were increased in NPY and DIO-NPY compared to their control groups suggesting insulin resistance. BIIE0246 increased insulin and HOMA-IR in chow fed groups but not in DIO.

**Table 1:** Serum glucose and cholesterol parametres.

	Chow diet			
	WT (*n* = 11–13)		NPY (*n* = 11–12)	
	Vehicle	BIIE0246	Vehicle	BIIE0246
Glucose (mM)	8.4 ± 0.6	8.0 ± 0.5	8.6 ± 0.4^P=0.05^	8.9 ± 0.3^P=0.05^
Insulin (μg l^-1^)	0.28 ± 0.03	0.44 ± 0.04^#^	0.47 ± 0.04^∗∗∗^	0.51 ± 0.06^∗∗∗#^
HOMA-IR	2.5 ± 0.3	3.6 ± 0.3^#^	4.3 ± 0.4^∗∗∗^	5.1 ± 0.5^∗∗∗#^
Cholesterol (mM)	2.3 ± 0.1	2.5 ± 0.1	2.6 ± 0.1^∗∗^	2.7 ± 0.1^∗∗^

	**Western diet**			
	**DIO-WT (*n* = 7–12)**		**DIO-NPY (*n* = 6–7)**	
	**Vehicle**	**BIIE0246**	**Vehicle**	**BIIE0246**

Glucose (mM)	9.3 ± 0.4	9.3 ± 0.6	9.4 ± 0.6	9.5 ± 0.6
Insulin (μg l^-1^)	0.75 ± 0.14	0.76 ± 0.10	1.03 ± 0.25^P=0.06^	1.51 ± 0.55 ^P=0.06^
HOMA-IR	7.6 ± 1.4	6.7 ± 0.9	9.8 ± 1.8^∗^	14.8 ± 4.8^∗^
Cholesterol (mM)	6.1 ± 0.4	6.4 ± 0.4	6.5 ± 0.5	5.8 ± 0.8

*The data is presented from a chow diet-fed cohort after 4.5 weeks of treatment and from a Western diet-fed cohort after 2 weeks of treatment with BIIE0246 or vehicle. Values are expressed as means ± SEM. ^∗^*P* < 0.05, ^∗∗^*P* < 0.01, ^∗∗∗^*P* < 0.001, *P* = 0.05 and *P* = 0.06 between the genotypes with two-way ANOVA. ^#^*P* < 0.05 comparing BIIE0246 treatment and vehicle treatment with two-way ANOVA. WT, wildtype mice on chow diet; NPY, OE-NPY^*D*β*H*^ mice on chow diet; DIO-WT, wildtype mice on Western diet; DIO-NPY, OE-NPY^*D*β*H*^ mice on Western diet.*

**FIGURE 3:**
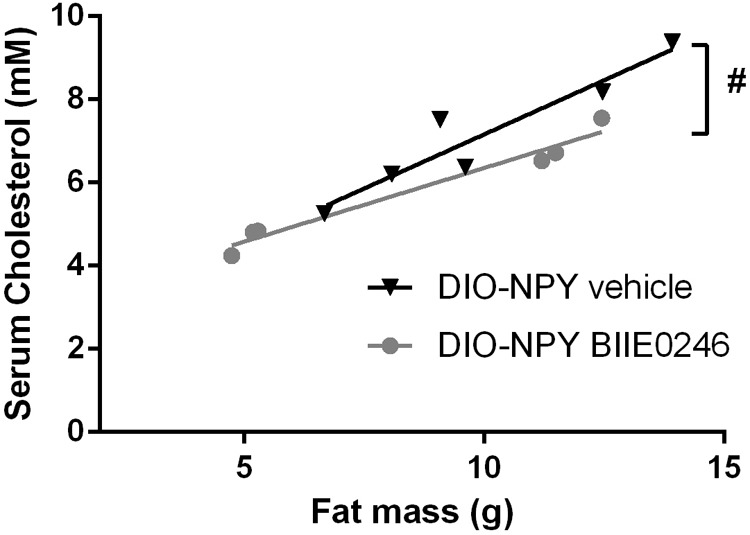
Positive correlation between serum cholesterol values and body fat mass and the difference between the correlation slopes in OE-NPY^DβH^ and WT mice on Western diet treated with Y_2_-receptor antagonist (BIIE0246) and vehicle for 2 weeks (*n* = 6–7/group). Values are expressed as means ± SEM. ^#^*P* < 0.05 comparing correlation curves between BIIE0246 and vehicle treatment with linear regression analysis. DIO-NPY, OE-NPY^DβH^ mice on Western diet.

Excess NPY increased serum cholesterol levels on healthy diet (**Table 1**). Interestingly, increased cholesterol levels were detected also in WT mice treated with BIIE0246 for 2 weeks (Supplementary Figure 3), but not in the 4.5-week cohort (**Table 1**). In DIO, BIIE0246 did not have significant effect on absolute cholesterol levels (**Table 1**). However, in DIO-NPY mice in both treatment groups, cholesterol levels correlated positively with body fat mass (DIO-NPY vehicle R^2^ = 0.90, *P* < 0.01; DIO-NPY BIIE0246 R^2^ = 0.96, *P* < 0.001), but not in any other group, and the slope of the regression curve of cholesterol and fat mass was significantly decreased in BIIE0246-treated DIO-NPY group when compared with vehicle-treated group (**Figure 3**). This suggests that serum cholesterol in DIO-NPY mice is highly dependent on body fat mass, and with similar body adiposity, serum cholesterol levels are lower after treatment with BIIE0246.

### Liver Weight and Morphology

**FIGURE 4:**
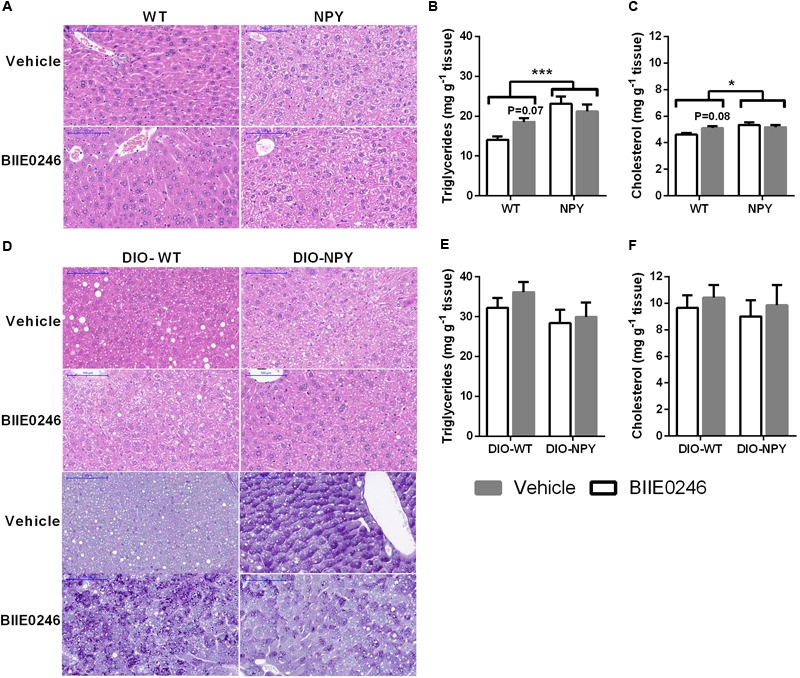
**(A)** Representative H&E stainings of liver slides with × 20 magnification (scale bar 100 μm), and **(B)** triglycerides and **(C)** cholesterol in the livers of mice on chow diet treated with Y_2_-receptor antagonist (BIIE0246) or vehicle for 4.5 weeks (*n* = 8–12/group). **(D)** Representative H&E (upper panel) and PAS stainings (lower panel) of liver slides with × 20 magnification (scale bar 100 μm), and **(E)** triglycerides and **(F)** cholesterol in the livers of mice on Western diet treated with Y_2_-receptor antagonist (BIIE0246) or vehicle for 2 weeks (*n* = 7–11/group). Values are expressed as means ± SEM. ^∗^*P* < 0.05 and ^∗∗∗^*P* < 0.001 comparing the different genotypes with two-way ANOVA. *P*-values present comparison of BIIE0246 with vehicle treatment within WT mice with Bonferroni *post hoc* test following a significant or near-significant interaction between treatment and genotype in two-way ANOVA. White bars, vehicle treated mice; gray bars, BIIE0246 treated mice; WT, wildtype mice on chow diet; NPY, OE-NPY^DβH^ mice on chow diet; DIO-WT, wildtype mice on Western diet; DIO-NPY, OE-NPY^DβH^ mice on Western diet.

Similarly to previous findings, NPY mice displayed ballooning degeneration and hepatic accumulation of triglycerides and cholesterol compared to WT mice on chow diet (**Figures 4A–C**). BIIE0246 had no influence on liver morphology (**Figure 4A**). However, it induced different responses between the genotypes in hepatic triglyceride and cholesterol levels (interaction *P* < 0.05 and *P* = 0.05, respectively) with a tendency to increased contents in WT mice and no change in NPY mice compared to vehicle treated mice (**Figures 4B,C**).

Also in DIO, ballooning degeneration was detected in vehicle-treated DIO-NPY mice compared with DIO-WT mice. Similarly, treatment with BIIE0246 induced ballooning degeneration in DIO-WT mice compared to vehicle-treated mice. Oppositely, in BIIE0246-treated DIO-NPY mice ballooning degeneration was less pronounced when compared with their vehicle controls (**Figure 4D**, upper panel). PAS staining revealed that the ballooning degeneration pointed to hepatic glycogen accumulation in vehicle-treated DIO-NPY and BIIE0246-treated DIO-WT mice, whereas in BIIE0246-treated DIO-NPY mice glycogen content was decreased (**Figure 4D**, lower panel). However, mRNA expressions of genes related to glycogen metabolism were not changed (data not shown). The hepatic contents of triglycerides and cholesterol were similar between the genotypes and the treatment groups (**Figures 4E,F**).

### Sympathetic and Central Noradrenergic Nervous System

**FIGURE 5:**
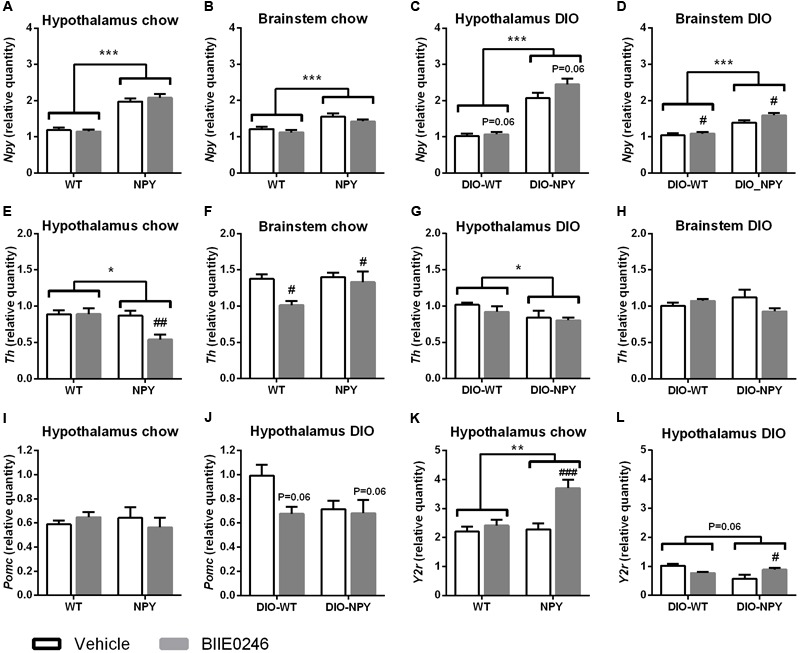
**(A–D)** mRNA expression of *Npy*, **(E–H)** *Th* **(I,J)** *Pomc* and **(K,L)** *Y2r* in the hypothalamus and the brainstem of chow- **(A,B,E,F,I,K)** or Western-diet-fed **(C,D,G,H,J,L)** OE-NPY^DβH^ and WT mice (*n* = 6–12/group) after 2-week Y_2_-receptor antagonist (BIIE0246) or vehicle treatment. Values are expressed as means ± SEM. ^∗^*P* < 0.05, ^∗∗^*P* < 0.01, ^∗∗∗^*P* < 0.001 and *P* = 0.06 comparing the different genotypes with two-way ANOVA, and ^#^*P* < 0.05, ^##^*P* < 0.01, ^###^*P* < 0.001 and *P* = 0.06 comparing BIIE0246 treatment and vehicle treatment with two-way ANOVA **(C,D,F,J)**, or with Bonferroni *post hoc* test following a significant interaction between treatment and genotype in two-way ANOVA **(E,K,L)**. *Npy*, Neuropeptide Y; *Pomc*, pro-opiomelanocortin; *Th*, Tyrosine hydroxylase; *Y2r*, Y_2_-receptor; white bars, vehicle treated mice; gray bars, BIIE0246 treated mice; WT, wildtype mice on chow diet; NPY, OE-NPY^DβH^ mice on chow diet; DIO-WT, wildtype mice on Western diet; DIO-NPY, OE-NPY^DβH^ mice on Western diet.

To elucidate the mechanisms of the metabolic effects of BIIE0246 mRNA levels of key genes in the sympathetic and central noradrenergic nervous systems were analyzed. The 2-week chow cohort was used in order to detect the primary effects of the drug rather than compensatory changes induced by weight change. First, the effect of BIIE0246 on peripheral NPY in SNS in adrenal glands was elucidated. As it did not have significant effect on the expression of *Npy* or *Y2r* in adrenal glands (data not shown), we next studied the central NPY and noradrenergic systems in the hypothalamus and the brainstem, the brain regions lacking an effective blood-brain barrier. First, the expression of *Th* and *Npy* were measured to study whether BIIE0246 directly or by regulating *Npy* expression could influence the production of noradrenaline. In DIO (but not on chow diet), BIIE0246 increased *Npy* expression in both genotypes (**Figures 5A–D**). Instead on chow diet (but not in DIO), BIIE0246 decreased *Th* expression in the hypothalamus of NPY mice and in the brainstem of both genotypes (**Figures 5E–H**), suggesting that on healthy diet, BIIE0246 directly decreases central sympathetic tone. On the other hand, fitting with the changes in *Npy* expression, blocking of Y_2_-receptors with BIIE0246 in DIO (but not on chow diet) tended (*P* = 0.06) to decrease hypothalamic *Pomc* expression, especially in DIO-WT mice (**Figures 5I,J**), suggesting that with excess calories, BIIE0246 directly potentiates the expression of *Npy*, which in turn inhibits *Pomc* expression. Second, the effect of Y_2_-receptor antagonism on hypothalamic autoinhibitory Y_2_-receptor was studied. BIIE0246 significantly increased *Y2r* expression in NPY and DIO-NPY mice, whereas it had no effect in WT and DIO-WT mice [treatment × genotype interaction *P* < 0.05 (chow) and *P* < 0.01 (DIO)] (**Figures 5K,L**).

### WAT and BAT on Western Diet

**FIGURE 6:**
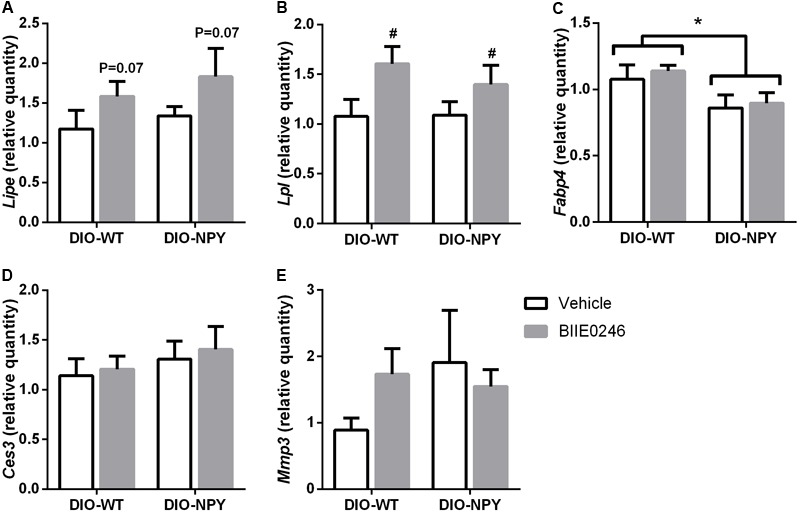
**(A)** mRNA expression of *Lipe*, **(B)** *Lpl*, **(C)** *Fabp4*, **(D)** *Ces3*, and **(E)** *Mmp3* in retroperitoneal WAT of Western-diet-fed OE-NPY^DβH^ and WT mice (*n* = 7–11/group) after 2-week Y_2_-receptor antagonist (BIIE0246) or vehicle treatment. Values are expressed as means ± SEM. ^∗^*P* < 0.05 comparing the different genotypes with two-way ANOVA. ^#^*P* < 0.05 and *P* = 0.07 treatment effect with two-way ANOVA with non-significant interaction genotype × treatment. *Lipe*, hormone sensitive lipase; *Lpl*, lipoprotein lipase; *Fabp4*, fatty acid binding protein 4; *Ces3*, carboxylesterase 3; *Mmp3*, matrix metallopeptidase; white bars, vehicle treated mice; gray bars, BIIE0246 treated mice; DIO-WT, wildtype mice on Western diet; DIO-NPY, OE-NPY^DβH^ mice on Western diet.

Last, to elucidate whether the inhibition of fat accumulation in DIO-NPY mice could be explained by direct effects of BIIE0246 on adipose tissue, mRNA expression of selected genes involved in adipogenesis and angiogenesis in retroperitoneal WAT (WAT/r) and thermogenesis in BAT were studied. Weights of WAT depots did not differ between the treatments (data not shown). In WAT/r the lipases *Lipe* and *Lpl* tended or were significantly increased by BIIE0246, respectively, without difference between the genotypes (**Figures 6A,B**). The adipogenic *Fabp4* was decreased in DIO-NPY compared to DIO-WT mice without difference between the treatments. Another adiopogenic marker, *Ces3*, and angiogenesis marker *Mmp3* did not differ between the treatments or the genotypes (**Figures 6C–E**). BAT weight or the expression of the thermogenesis marker *Ucp1* in BAT were not changed by BIIE0246 (data not shown).

## Discussion

Genetic down-regulation of peripheral Y_2_-receptors has previously been shown to inhibit NPY’s ability to reduce energy expenditure and to increase WAT mass (Kuo et al., 2007; Shi et al., 2011). In this study, we elucidated whether pharmacological blockage of peripheral Y_2_-receptors with BIIE0246 would have beneficial effects on energy metabolism in a situation of excess energy and/or NPY, usually associated with obesity (Shi et al., 2013). This study confirms the finding that blocking of Y_2_-receptors has beneficial effects on fat deposition and the metabolic status, but only when energy-rich environment is combined with excess NPY. Instead with normal NPY and excess calories, an Y_2_-receptor antagonist induces obesity. On a healthy diet, a peripherally administered BIIE0246 increases body weight gain and causes metabolic disturbances, especially when NPY levels are normal.

Y_2_-receptor antagonism-induced reduction in fat mass gain in the situation of elevated NPY levels and energy surplus, i.e., in DIO-NPY mice, fits with the findings by Kuo et al. (2007). They showed that chronic stress-induced NPY release from SNS in combination with energy-rich diet promoted fat mass gain via direct effects on adipose tissue, and that this was prevented with adipose tissue-targeted treatment with BIIE0246. NPY was suggested to induce fat mass gain directly by stimulating the proliferation and differentiation of adipocytes, and indirectly by promoting angiogenesis (Kuo et al., 2007). Although in our study BIIE0246 did not induce significant differences in the gene expressions of adipogenic and angiogenic genes in the retroperitoneal WAT, it does not exclude the fact that this is the most probable mechanism-of-action in inhibition of fat mass gain in presence of excess NPY (i.e., in DIO-NPY mice). Instead, BIIE0246 increased the expression of enzymes responsible for both triglyceride uptake and breakdown suggesting that the accelerated lipid turn-over may influence the outcome in both genotypes. The catabolic effect of BIIE0246 in DIO-NPY mice was also reflected in decreased hepatic glycogen accumulation and serum cholesterol relative to body weight suggesting a role for Y_2_-receptors in regulation of these traits in a state of excess NPY and energy surplus. This fits with the improved serum cholesterol in Y_2_-receptor antagonist treated diabetic rats (Liu et al., 2013), which have also been shown to have elevated NPY levels (Morris and Pavia, 2004; Ruipan et al., 2014).

In contrast, without excess peripheral NPY (i.e., in DIO-WT mice) treatment with BIIE0246 does not lead to beneficial effects but rather increases fat and glycogen accumulation suggesting different mechanisms depending on the level of NPY. This difference between genotypes supports that Y_2_-receptor plays a key role in mediating the obesogenic effects of NPY in WAT in situations of NPY excess due to stress (Kuo et al., 2007) or genetic overexpression as presented here. The induction of metabolic disturbances is even more prominent when the mice are fed with a healthy diet. In chow-fed WT mice, BIIE0246 increased weight gain, and caused hypercholesterolemia, hyperinsulinemia, and hepatic triglyceride and cholesterol accumulation, which actually resemble the metabolic phenotype of genetically obese NPY mice (Vahatalo et al., 2015; Ailanen et al., 2017). These findings in WT and DIO-WT mice are opposite to the results of peripheral Y_2_-receptor knock-down that induced resistance to DIO and had no effects on chow (Shi et al., 2011). The discrepancy in the results could be influenced by the method of receptor inhibition, i.e., by the stress induced by the drug administration and the fluctuation in drug concentration over the day due to once daily dosing combined with the relatively short half-life of BIIE0246. However, even more likely it could result from the effects of neural Y_2_-autoreceptor inhibition that was avoided by the knock-down approach.

Therefore, we hypothesized that antagonism of presynaptic Y_2_-autoreceptors increased NPY release in WT mice. As no differences in the expression of *Npy* or *Y2r* were detected in the adrenal glands, the central effects of Y_2_-receptor antagonism were considered. As the hypothalamus and brainstem are some of the rare brain regions that lack an effective blood-brain barrier (Wang et al., 2008; Rodriguez et al., 2010), peripherally administered BIIE0246 could have reached Y_2_-receptors in these key areas regulating energy balance. NPY neuron-specific Y_2_-receptor deletion in the hypothalamus has been shown to increase *Npy* and decrease *Pomc* expression in the Arc (Shi et al., 2010), and *Npy* overexpression in the Arc to increase food intake and to inhibit SNS activity via decreased expression of hypothalamic *Th*, the rate-limiting enzyme of catecholamine synthesis (Shi et al., 2013). Brainstem *Npy* overexpression in turn downregulates brainstem *Th*, but does not increase food intake (Vahatalo et al., 2015). In the current study, a small increase in hypothalamic and brainstem *Npy*, and a tendency to decreased *Pomc* were detected, but only in the DIO group. This could have contributed to the obesogenic effect of BIIE0246 in mice with normal NPY levels, although it did not affect food intake or *Th* expression. Instead, in chow-fed mice, no changes in *Npy* expression or food intake were detected, but brainstem *Th* was downregulated and thus lower SNS activity could play a role in the metabolic disturbances induced by BIIE0246 similar to genetic *Npy* overexpression in NPY mice (Vahatalo et al., 2015). Interestingly, BIIE0246 changed *Y2r* expression differently between the genotypes. Hypothalamic *Y2r* expression was increased in NPY mice treated with BIIE0246 on both diets. This could have contributed to somewhat milder adverse metabolic effect in NPY mice compared to WT mice, and to the beneficial metabolic effect in DIO-NPY mice compared to DIO-WT. Thus, in addition to the blockage of peripheral Y_2_-receptors, it is likely that hypothalamic and brainstem Y_2_-receptor antagonism contributed to the metabolic outcomes in the current study. Central effects are also supported by the resemblance of the BIIE0246-induced phenotype in WT mice with the obese phenotype of adult-onset hypothalamic Y_2_-receptor knock-out mice (Shi et al., 2013).

Taken together, the peripheral Y_2_-receptors are clearly participating in the regulation of energy metabolism, but whether their pharmacological blockage is beneficial in the treatment of obesity and metabolic disorders, remains even more controversial than previous studies have suggested. Although we cannot exclude the fact that the effect of the incomplete antagonism of Y_2_-receptors due to the fluctuation of the drug concentration may influence the results of this study, it seems that on a regular chow diet, independent of normal or excess NPY, Y_2_-receptors accessible to a peripherally administered Y_2_-receptor antagonist seem to be beneficial to energy metabolism, and their blockage leads to weight gain and metabolic disorders possibly via centrally decreased sympathetic activity. In an energy-rich environment, pharmacological Y_2_-receptor blockage is metabolically beneficial supporting the key role of Y_2_-receptor in expanding adipose tissue. However, this is true only when NPY levels are increased. In humans, elevated NPY levels are associated with increased risk for metabolic disorders in individuals with gain-of-function polymorphism in the *NPY* gene (Kallio et al., 2001; Ding et al., 2005; Yeung et al., 2011). Thus, since the trend in medical care is moving toward more personalized pharmacotherapy, the blockade of peripheral Y_2_-receptors could be targeted for the treatment of the obesity accompanied with elevated noradrenergic NPY levels (e.g., during stress or genetic *NPY* overexpression) in energy rich environment. In this sense, OE-NPY^DβH^ mice could be a potential tool for the pharmacological studies of Y_2_-receptor specific compounds.

## Author Contributions

LV and LA conceived and performed the study, analyzed the data, and wrote the manuscript with equal contributions. HS-M, SM, and WO took part in performing the study. SR and ES conceived the study and wrote the manuscript.

## Conflict of Interest Statement

The authors declare that the research was conducted in the absence of any commercial or financial relationships that could be construed as a potential conflict of interest.
