# Supplementary material for: Peripherally Administered Y2-Receptor Antagonist BIIE0246 Prevents Diet-Induced Obesity in Mice With Excess Neuropeptide Y, but Enhances Obesity in Control Mice
Source: Front Pharmacol. 2018 Apr 5;9:319. doi: 10.3389/fphar.2018.00319 (PMC5895854; doi:10.3389/fphar.2018.00319)
Supplement: Supplementary file 1 [file Data_Sheet_1.docx]

Supplementary Material

**Peripherally administered Y_2_-receptor antagonist BIIE0246**

**prevents diet-induced obesity in mice with excess neuropeptide Y,**

**but enhances obesity in control mice**

**Liisa Ailanen, Laura H. Vähätalo, Henriikka Salomäki-Myftari, Satu Mäkelä, Wendy Orpana, Suvi T. Ruohonen, and Eriika Savontaus^*^**

**^*^Correspondence:** Eriika Savontaus: eriika.savontaus@utu.fi

**Supplementary Figure 1.** Study protocol for blocking of Y_2_-receptors with BIIE0246 of OE-NPY^DβH^ and WT mice on chow or Western diet

**Supplementary Figure 2. (A-B)** Baseline body weight, **(C-D)** fat mass, **(E-F)** lean mass and **(G-H)** average daily energy intake per cage in OE-NPY^DβH^ and WT mice subjected to chow (n=19-25 mice / group in A, C and E, and n=7-10 cages / group in G) or Western diet (n=7-11 mice / group in B, D and F, and n=3-4 cages / group in H) prior to drug treatments. Values are expressed as means ± SEM. *P<0.05 and ***P<0.001 comparing different genotypes with two-way ANOVA, when treatment x genotype interaction was non-significant. White bars = vehicle-treated group, grey bars = BIIE0246 treated group. WT = wildtype mice on chow diet, NPY = OE-NPY^DβH^ mice on chow diet, DIO-WT= wildtype mice on Western diet, DIO-NPY= OE-NPY^DβH^ mice on Western diet.

**Supplementary Figure 3. (A)** Body weight, **(B)** fat mass and **(C)** lean mass gain, **(D)** fasting blood glucose and **(E)** serum cholesterol in OE-NPY^DβH^ and WT mice (n=11-12/group) on chow diet treated with Y_2_-receptor antagonist (BIIE0246) or vehicle for 2 weeks. Values are expressed as means ± SEM. *P<0.05 comparing the different genotypes with two-way ANOVA, and ^#^P<0.05, ^##^P<0.01 and P=0.07 comparing BIIE0246 treatment and vehicle treatment with two-way ANOVA (A, C), or with Bonferroni post-hoc test following a significant interaction between treatment and genotype in two-way ANOVA (E). White bars = vehicle treated mice, grey bars = BIIE0246 treated mice, WT = wildtype mice on chow diet, NPY = OE-NPY^DβH^ mice on chow diet.
